# Postpartum depression and major depressive disorder: the same or not? Evidence from resting-state functional MRI

**DOI:** 10.1093/psyrad/kkac015

**Published:** 2022-11-21

**Authors:** Bochao Cheng, Yi Guo, Xijian Chen, Bin Lv, Yi Liao, Haibo Qu, Xiao Hu, Haoxiang Yang, Yajing Meng, Wei Deng, Jiaojian Wang

**Affiliations:** Department of Radiology, West China Second University Hospital of Sichuan University, Chengdu 610041, China; Key Laboratory of Birth Defects and Related Diseases of Women and Children (Sichuan University), Ministry of Education, Chengdu 610041, China; Department of Radiology, West China Second University Hospital of Sichuan University, Chengdu 610041, China; Department of Radiology, West China Second University Hospital of Sichuan University, Chengdu 610041, China; Department of Gynecology and Obstetrics, West China Second University Hospital, Sichuan University, Chengdu 610041, China; Department of Radiology, West China Second University Hospital of Sichuan University, Chengdu 610041, China; Department of Radiology, West China Second University Hospital of Sichuan University, Chengdu 610041, China; Department of Rehabilitation Medicine, West China Second University Hospital, Sichuan University, Chengdu 610041, China; Department of Radiology, West China Second University Hospital of Sichuan University, Chengdu 610041, China; Department of Psychiatry, West China Hospital of Sichuan University, Chengdu 610041, China; Department of Psychiatry, West China Hospital of Sichuan University, Chengdu 610041, China; Affiliated Mental Health Center & Hangzhou Seventh People's Hospital, Zhejiang University School of Medicine, Hangzhou, Zhejiang 310063, China; State Key Laboratory of Primate Biomedical Research, Institute of Primate Translational Medicine, Kunming University of Science and Technology, Kunming 650500, China; Yunnan Key Laboratory of Primate Biomedical Research, Kunming, Yunnan 650500, China

**Keywords:** postpartum depression, major depressive disorder, resting-state fMRI, functional activity, functional connectivity

## Abstract

**Background:**

Although postpartum depression (PPD) and non-peripartum major depressive disorder (MDD) occurring within and outside the postpartum period share many clinical characteristics, whether PPD and MDD are the same or not remains controversial.

**Methods:**

The current study was devoted to identify the shared and different neural circuits between PPD and MDD by resting-state functional magnetic resonance imaging data from 77 participants (22 first-episodic drug-naïve MDD, 26 drug-naïve PPD, and 29 healthy controls (HC)).

**Results:**

Both the PPD and MDD groups exhibited higher fractional amplitude of low-frequency fluctuation (fALFF) in left temporal pole relative to the HC group; the MDD group showed specifically increased degree centrality in the right cerebellum while PPD showed specifically decreased fALFF in the left supplementary motor area and posterior middle temporal gyrus (pMTG_L), and specifically decreased functional connectivities between pMTG and precuneus and between left subgeneual anterior cingulate cortex (sgACC_L) and right sgACC. Moreover, sgACC and left thalamus showed abnormal regional homogeneity of functional activities between any pair of HC, MDD, and PPD.

**Conclusions:**

These results provide initial evidence that PPD and MDD have common and distinct neural circuits, which may facilitate understanding the neurophysiological basis and precision treatment for PPD.

## Introduction

Postpartum depression (PPD) is the most likely mental disorder for women after childbirth, with the core symptoms comprising unhappiness, irritability, fatigue, anxiety, paranoia, and depressed mood (Field, 2017). Regardless of its precise prevalence rate, PPD presses a significant public health issue not only on the lives of the women themselves, but also on their children's development (Drury *et al*., 2016; Glasheen *et al*., 2010). Although the devastating sequences of PPD have been well studied, the accurate category of PPD is not well-defined. The Diagnostic and Statistical Manual of Mental Disorders, Fifth Edition (DSM-5) defines PPD as a major depressive disorder (MDD) occurring during pregnancy or 4 weeks peripartum onset. Other organizations such as the World Health Organization (WHO) defines the PPD onset within 1 year after postpartum (Stewart *et al*., 2008). In spite of non-peripartum MDD being a strong predictor of PPD, PPD and MDD present not only similar but also different clinical characteristics (O'Hara and McCabe, 2013), PPD seems not a simple subtype but maybe a distinct disorder. Except for the special time of onset, PPD mothers are easily irritated by their babies, respond less sensitively to, and more negatively to their babies relative to healthy mothers (Bloch *et al*., 2003). Both human and animal studies point out that fluctuations of reproductive hormone levels after childbirth may contribute to PPD (Bloch *et al*., 2000; Suda *et al*., 2008). Besides, PPD is more susceptible to resistance to conventional antidepressants (Hendrick *et al*., 2000), and presents a higher suicidal tendency than non-peripartum MDD (Stein *et al*., 2018).

Increasing functional magnetic resonance imaging (fMRI) evidence also supports the view that PPD and MDD are distinct disorders. Using resting-state fMRI (rs-fMRI), PPD patients typically show hypoactivity in both cortical and limbic structures (i.e. amygdala and hippocampus), while nonparturient MDD patients exhibit hypoactivity in more lateral cognition-related areas and hyperactivity in medial affective and subcortical limbic regions (Alcaro *et al*., 2010; Northoff *et al*., 2011). In addition, non-postpartum MDD patients exhibit hypoactivity in amygdala while watching happy emotional faces and increased amygdala activation when viewing negative faces (Groenewold *et al*., 2013), whereas PPD patients show a hypoactivity of amygdala in watching negative emotional faces (Moses-Kolko *et al*., 2010). However, these results are not obtained directly by comparing the two disorders. Therefore, the detailed information about common or distinct neural mechanisms underlying PPD and MDD is still unclear.

To disentangle the common or distinct neural circuits for PPD and MDD, the functional activity, local information integration, global functional integration, and long-distal functional connectivities (FCs) characterized using fractional amplitude of low frequency fluctuations (fALFF) (Zang *et al*., 2007), regional homogeneity (ReHo) (Zang *et al*., 2004), weighted degree centrality (DC) (Liu *et al*., 2018a; Song *et al*., 2019; Wu *et al*., 2016), and FC were analyzed between healthy controls (HC), PPD, and MDD. First, the voxel-level fALFF, ReHo, and DC maps for PPD, MDD, and HCs groups were calculated. The analyses of variance (ANOVA) were then applied to identify the divergence among the three groups. Then a seed-based FC analysis was applied using the regions with abnormal fALFF, ReHo, or DC as seeds to discover abnormal intrinsic FC. Correlation analyses were further used to check the relationship between changed neural measurement and clinical performance.

## Materials and Methods

### Participants

The data were collected based on a longitudinal project investigating the pathogenesis of PPD in Chengdu, China (Cheng *et al*., 2022a; Cheng *et al*., 2022b; Cheng *et al*., 2022c). From 1 June 2018 to 1 January 2020, seven-eight right-handed, age-matched women including 22 first-episode, treatment-naïve patients with MDD, 26 treatment-naïve patients with PPD, and 29 healthy controls (HC) were enrolled at the West China Second University Hospital (visits within 1 year postpartum, ages from 21 to 42 years). The diagnosis of PPD or MDD was performed based on the criteria of the Diagnostic and Statistical Manual of Mental Disorders, Fifth Edition (DSM-5) and the Chinese Classification and Diagnostic Criteria of Mental Disorders Third edition (CCMD-3) by two experienced psychiatrists (WD and YJ). The PPD and MDD were evaluated with Edinburgh Postnatal Depression Scale (EPDS) and Hamilton Rating Scale for Depression (HRSD), respectively. All patients experienced their first episode of depressive symptoms, and were treatment-naïve, right-handed, matched with age and education level. All the PPD patients have full-term, regular puerperium, and healthy infants. Participants with medical diseases such as cardiovascular diseases, diabetes, obvious anxiety symptoms (Beck Anxiety Inventory >45), history of depression, and any other Axis I mental disorders, substance dependence, history of attempted suicide, vasoactive medications, or MRI contradiction were excluded. The exclusion criteria for postpartum women includes adverse obstetric history [premature delivery (delivery <37 weeks), infants of low-birth weight (weight < 2.5 kg), dystocia], infants with any congenital disease. The demographic characteristics of the participants are summarized in Table 1. Written informed consents were collected from all participants. This study was authorized by the local ethics committee of West China Second University Hospital of Sichuan University (No. 106) followed by the Helsinki Declaration.

**Table 1:** Demographic and clinical characteristics.

	HC	MDD	PPD	*P* value
Number of participants	29	22	26	-
Age (mean ± SD)	32.48 ± 11.04	37.55 ± 11.5	34.5 ± 2.21	0.16
Gender (male/female)	0/29	0/22	0/26	1
FD_Power	0.12 ± 0.074	0.098 ± 0.045	0.12 ± 0.056	0.3
Depression scale (mean ± SD)	-	HRSD scores	EPDS scores	-
	-	23.95 ± 4.64	17.46 ± 3.55	-

Note: The one-way ANOVA was used for age and FD_Power comparisons. FD: frame-wise displacement; HRSD: Hamilton Rating Scale for Depression. EPDS: Edinburgh Postnatal Depression Scale.

### MRI data acquisition

The rs-fMRI data were collected on a Siemens 3.0 T MRI system with a 32-channel head coil (MAGNETOM Skyra, Siemens Medical Solutions, Erlangen, Germany), using a standard echo planar imaging (EPI) sequence. The foam padding and earplugs were used to minimize head motion and to muffle scanner noise. Participants were requested to relax, remain awake with their eyes closed, and avoid thinking during the MRI acquisition. Resting-state functional images were collected with a repetition time = 3.05 s, echo time = 22.5 ms, flip angle = 30°, 36 slices, thickness/gap = 4.0/1 mm, voxel size = 2.45 × 2.45 × 4 mm^3^, matrix size = 94 × 94, and field of view = 230 × 230 mm^2^.

### Rs-fMRI data preprocessing

The data preprocessing process is as follows: discard the first six volumes; realign all the images to the first volume; normalize all the images to the Montreal Neurological Institute (MNI) EPI template and resample at 3 × 3 × 3 mm^3^; and regress out the Friston 24-parameter model of head motion, white matter, cerebrospinal fluid, and global mean signals. The fractional amplitude of low-frequency fluctuation (fALFF) was calculated for each participant. Next, the functional images were filtered with a temporal band path of 0.01–0.1 Hz. Participants with a displacement of more than 2.5 mm and an angular motion of more than 2.5° were excluded to exclude head motion effects. No participants were excluded from this criterion. Moreover, the scrubbing method with cubic spline interpolation was further used to eliminate the bad images exceeding the preset criteria[ frame-wise displacement (FD) < 0.5] for excessive motion. Finally, the ReHo of functional activities and DC were calculated for each participant.

For FC connectivity, fMRI data were normalized to MNI space, then smoothed with a Gaussian kernel of 8 mm, Friston 24-parameter model of head motion, white matter, cerebrospinal fluid, and global mean signals were regressed out, and were filtered with a temporal band-path of 0.01–0.1 Hz, and scrubbed with cubic spline interpolation.

### fALFF, ReHo, and DC calculation

The fALFF, ReHo, and DC were used to measure the amplitude of low frequency oscillations, similarity or synchronization between the time series of a designated voxel and its nearest neighbors, along with functional integration or FC strength for a specific voxel of the brain, respectively. To calculate fALFF, the time series of each voxel was transformed to the frequency domain using a fast Fourier transform and the square root of each frequency was calculated and averaged across 0.01–0.1 Hz. The fALFF is defined as the power within 0.01–0.1 Hz divided by the total power of the full frequency range. ReHo is defined by calculating the Kendall's coefficient concordance of a time series of a given voxel to its neighboring 26 voxels. To calculate DC, the Pearson's correlation coefficients between the time series of a specific seed voxel and that of the rest of the brain were calculated, and a threshold of 0.25 was applied to remove weak connections that may arise from signal noise. The DC was calculated by averaging the correlation coefficients higher than this threshold over the whole brain. Finally, the fALFF, ReHo, and DC maps were converted to *z* scores to improve normality. The *z*-score maps of fALFF, ReHo, and DC were spatially smoothed with 8 mm full-width at half maximum for statistical purposes.

### Seed-based FC analyses

Resting-state FC analysis was performed to further detect whether the brain areas with differences in fALFF, ReHo, or DC showed altered functional couplings with the rest of the of brain. The Fisher's *z* transformation was applied to transfer FC value to a *z* value for statistical analyses.

### Statistical analyses

The analysis of variance (ANOVA) was applied with age and head motion parameters as covariates to check the differences in fALFF, ReHo, DC, and FC. Significance level was set at *P* < 0.05 corrected with false discovery rate (FDR) method (cluster size >50 voxels). To determine between-group difference, the mean fALFF, ReHo, DC, and FC were calculated and two-sample *t*-tests were used. The significance level was corrected using FDR with *P* < 0.05.

### Correlation analysis

To investigate whether the altered fALFF, ReHo, DC, or FC was related to the EPDS or HRSD scores, the correlation analyses were performed and the significance level was set at *P* < 0.05 (FDR corrected).

## Results

### Demographic and clinical characteristics

The PPD, MDD, and HC groups were well-matched in age (PPD: 34.5 ± 2.21, MDD: 37.55 ± 11.5, HC: 32.48 ± 11.04, *F* = 1.88, *P* = 0.11). The mean EPDS score for PPD is 17.46 ± 3.55 and the mean HRSD score for MDD is 23.95 ± 4.64 (see Table 1).

### Differences in fALFF, DC, and ReHo

**Figure 1:**
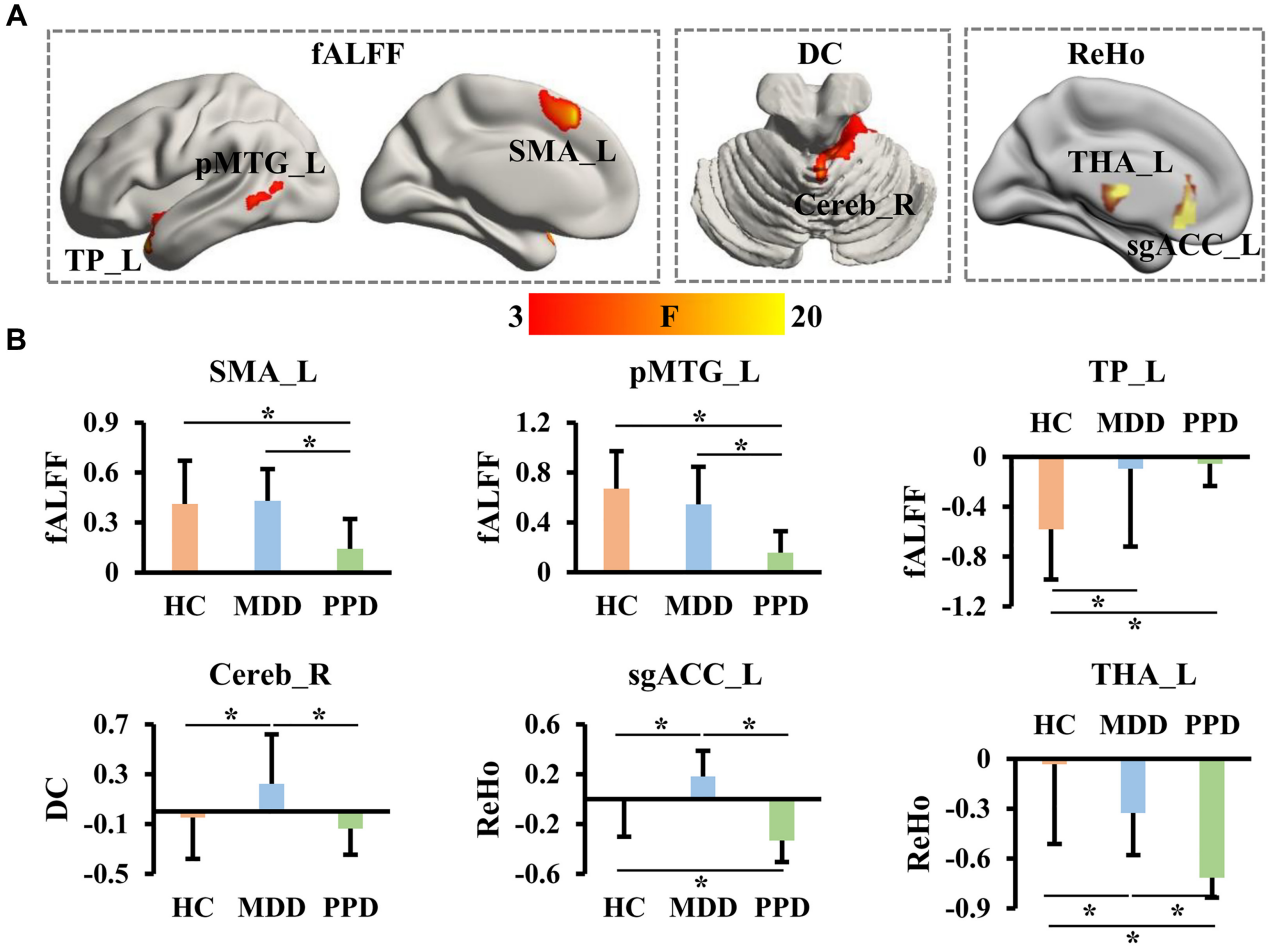
Differences in fALFF, DC, and ReHo among PPD, MDD, and HC. (A). One-way analysis of variance for fALFF, DC, or ReHo map was performed and significant differences found in left temporal pole (TP_L), pMTG_L, and left supplementary motor area (SMA_L) among PPD, MDD, and HC. (B). Post hoc two-sample *t*-tests analyses were further used to determine the between group differences in fALFF, DC, and ReHo.

The ANOVA observed significant differences in fALFF in left temporal pole (TP_L), left posterior middle temporal gyrus (pMTG_L), and left supplementary motor area (SMA_L) (Fig. 1A and Table 1). The significant difference in DC among PPD, MDD, and HC was found in the right cerebellum (Cereb_R) (Fig. 1A and Table 1). Furthermore, PPD, MDD, and HC demonstrated significant differences in ReHo in left thalamus (THA_L) and subgenual anterior cingulate cortex (sgACC_L) (Fig. 1A and Table 2).

**Table 2:** Changed fractional amplitude of fALFF, DC, ReHo, and FC among HC, MDD, and PPD disorder.

Brain regions	Activity	*F* value	Primary peak coordinate (mm)		
**fALFF:**					
TP.L	increase	25.91	−45	12	−30
pMTG.L	decrease	24.42	−57	−66	15
SMA.L	decrease	26.15	−3	27	51
**DC:**					
Cereb.R	increase	14.66	12	−48	−21
**ReHo:**					
SgACC.L	increase	11.18	−15	18	−6
THA.L	increase	10.86	−15	−15	3
**FC:**					
pMTG_L-Pcu.B	decrease	9.68	0	−54	42
sgACC.L-sgACC.R	decrease	12.95	15	18	−9

MNI: Montreal Neurological Institute; L: left hemisphere; R: right hemisphere; TP: temporal pole; Cereb: cerebellum; THA: thalamus; L: left; R: right; B: bilateral.

Post hoc two sample *t*-test analyses found that PPD had specifically decreased fALFF in SMA_L and pMTG_L relative to both MDD and HC, while no difference between MDD and HC in the two areas was found (Fig. 1B). Both PPD and MDD exhibited greater fALFF in TP_L compared to HC but no difference between PPD and MDD (Fig. 1B). MDD showed specifically increased DC in Cereb_R compared to both PPD and HC, and PPD and HC showed no significant difference (Fig. 1B). In THA_L and sgACC_L, there were significant differences in ReHo between any pair of PPD, MDD, and HC (Fig. 1B). In particular, PPD had more ReHo in sgACC_L and THA_L than MDD and HCs, and MDD had more ReHo in sgACC_L and THA_L than HCs (Fig. 1B & Table 2).

### Changed FCs

**Figure 2:**
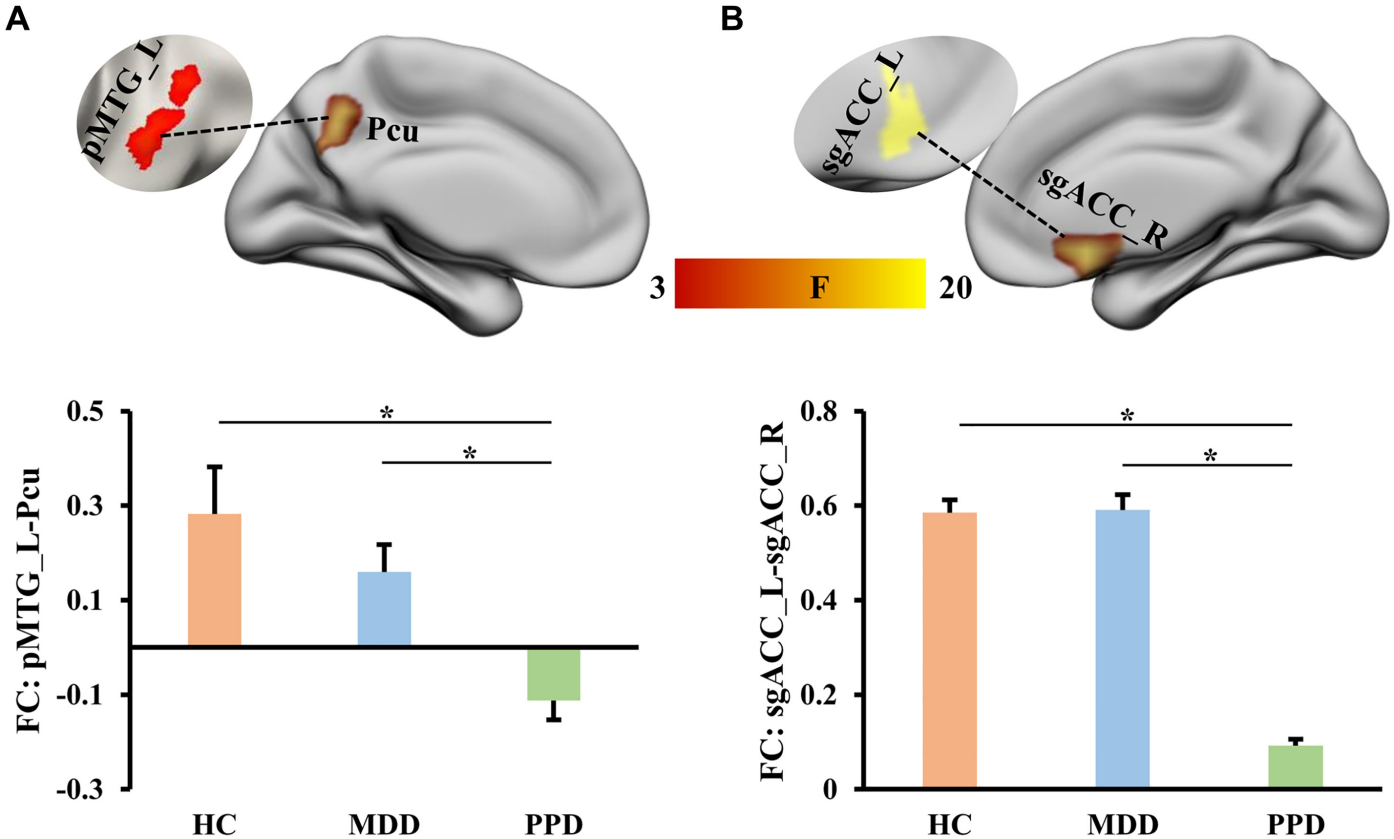
Differences in resting-state FC among PPD, MDD, and HC. One-way analysis of variance for FC was performed and found significant differences between pMTG_L and Pcu and between left and right sgACC among PPD, MDD, and HC.

Seed-based FC analyses revealed significant differences in functional couplings between pMTG_L and precuneus (Pcu), and between sgACC_L and right sgACC (Fig. 2). For both FCs of pMTG_L with Pcu and sgACC_L with right sgACC, PPD showed specifically reduced FCs relative to both MDD and HC while no significant differences between MDD and HC were identified (Fig. 2).

### Correlation analyses

No significant correlations were found between altered neural measures and EPDS or HRSD scores in our study.

## Discussion

The current results confirm our hypothesis that there are common and disorder-specific neuromarkers between first-episode, treatment-naïve PPD and MDD. PPD and MDD groups shared increased fALFF in TP_L compared to HC. As for the disorder-specific neuromarkers, PPD had specifically decreased fALFF in SMA_L and pMTG_L, while MDD showed specifically increased DC in Cereb_R, compared to the other two groups, respectively. Interestingly, distinct differences of ReHo were identified between any pair of the three groups in THA_L and sgACC_L. For the functional coupling, PPD showed specifically reduced FC between pMTG_L-Pcu and between sgACC_L-sgACC.R compared to both MDD and HC.

We first observed both PPD and MDD shared the increased fALFF in TP_L relative to HC. The temporal lobe is a complex region for advanced brain function activities including memory retention and emotion regulation. Some studies supposed a distorted moduation of inner and outer information in depressive patients, due to the dysfunction of temporal cortices (Xiao-juan *et al*., 2011). These abnormalities involve emotion regulation, memory processing, and social cognition (Beauregard *et al*., 2006), consisting of reduced GMV (Bora *et al*., 2012), decreased regional activity, and abnormal FC (Guo *et al*., 2012; Wu *et al*., 2013). More specifically, TP participates in visceral emotion modulation when perceptually stimulated (Kondo *et al*., 2003), and participants in emotional self-regulation, which is related to emotion dysregulation in depression (Beauregard *et al*., 2006). TP damage can affect mood states; its volume is inversely correlated with the depressive symptoms (Glosser *et al*., 2000; Zorzon *et al*., 2002). Therefore, the increased fALFF in TP_L for both MDD and PPD illustrates a common disrupted neural activity concerning emotion dysregulation in depression subtypes.

Importantly, our results identified several distinct brain functions and conectomes between PPD and MDD, including decreased fALFF in SMA_L and pMTG_L of PPD, increased DC of Cereb_R in MDD, and abnormal ReHo between any pair of three groups in THA_L and sgACC_L, respectively. PPD showed specifically reduced FC between pMTG_L-Pcu and between sgACC_L-sgACC_L. pMTG was reported to be primarily associated with language and social cognition processing (Xu *et al*., 2019; Xu *et al*., 2015). pMTG has been condemned as a node of dorsal attention and default mode network (DMN) (Fox *et al*., 2006; Wang *et al*., 2012), and involves emotional face recognition (Khalaf *et al*., 2016). Increased local ReHo and reduced GMV, ALFF, and functional couplings with other brain regions were recognized in pMTG of depressive patients (Guo *et al*., 2012; Karim *et al*., 2017; Liu *et al*., 2018b; Peng *et al*., 2011; Wu *et al*., 2011), and a decreased ReHo was reported in remitted geriatric depression (Yuan *et al*., 2008). Our recent study found the decreased FC in sgACC-pMTG in PPD patients, which exhibited a negative correlation with PPD loads (Cheng *et al*., 2022c). These findings collectively indicate the structural and functional abnormalities in pMTG are a trait alteration that underlines PPD.

As a core node of the DMN, Pcu has been implicated in internally directed attention and thoughts (Leech *et al*., 2011), involving self-referential mentation (Sestieri *et al*., 2011). Deficits in self-referential processes such as self-criticism (Hartlage *et al*., 1998) with altered FCs within DMN were thought to be a trait of current depression. Midline structures comprise the medial frontal gyrus, anterior cingulate cortex (ACC), posterior cingulate cortex (PCC), and Pcu, and are involved in imagery and self-referential processes, reflecting personal memory retrieval (Macdonald *et al*., 2016). Reduced activation in these regions was found in remitted depression while attempting to repair a sad mood with positive autobiographical memories, accompanied by lower activation predicting the recurrence (Macdonald *et al*., 2016). Although it is unclear whether there is a direct link between the pMTP and Pcu, a lot of studies have reported hypoactivity in Pcu in depressive patients (Guo *et al*., 2013), and lower FC between MTG and Pcu (Yan *et al*., 2022). Thus, compared with MDD, PPD have a decreased fALFF in pMTG and lower FC in pMTG-Pcu suggest a dysregulated DMN with worse emotion control and deficits in positive memory retrieval.

As is known, both SMA and cerebellum are key nodes of mirror neuron system (MNS), participating in social cognition and interaction (Kato and Kato, 2014). It is worth recognizing that mother–infant interaction concerns social interaction, integrating the simulation of motor action and higher cognitive functions so as to require inferences about mental states (Spunt and Lieberman, 2012). In social cognition, the MNS and the mentalizing network are commonly distinguished (Van Overwalle, 2009; Van Overwalle and Baetens, 2009). The mother shows greater activations in the mentalizing network in response to the synchronous social cues, and deactivation of this network was comparatively more robust in depressive cases (Atzil *et al*., 2014). Our results indicate that PPD has lower fALFF in SMA than MDD, confirming our previous reports again that the suppressed MNS may account for the hampered social cognition in PPD patients (Cheng *et al*., 2022b). Thus, the abnormal mother–infant interaction, a sign of PPD, may implicate chaos in synergy of the MNS and mentalizing network.

Substantial evidence supports the prominent role for the cerebellum in social cognition via its projection to prefrontal and other regions (e.g. parietal and temporal cortices) (Reeber *et al*., 2013). Emotion is closely related to social cognition. Sufficient social interaction requires one to understand the emotions of others, including reading facial expressions, which are mediated by the cerebellum (Van Overwalle, 2009). Lesions of the cerebellum have been linked with reduced pleasure in response to positive stimuli (Turner *et al*., 2007), and this may produce emotional passivity (Schmahmann and Sherman, 1997). Notably, hyperactivity in the cerebellum on viewing negative emotional pictures (Groenewold *et al*., 2013) and increased FC strength in the cerebellum were reported in MDD patients (Murrough *et al*., 2016). On the contrary, PPD patients manifested a decreased FC strength in the cerebellum, which is negatively associated with the symptoms of PPD (Cheng *et al*., 2022b). Different from PPD, enhanced DC values in the cerebellum might imply a mild impairment of social cognitive function in MDD.

Moreover, we found the prominent differences of ReHo in THA_L and sgACC_L between any pair of HC, PPD, and MDD groups. Particularly, ReHo values in sgACC_L sequentially decreased from MDD to PPD to HC, while ReHo values in THA_L sequentially decreased from PPD to MDD to HC. As well as being well known as a relay station for sensory information transmission, THA plays significant roles in emotion, memory, arousal, etc. Besides, THA participates in the process of attention control through cortico-limbic circuits, and thus affects cognition (Halassa and Kastner, 2017). There are two pathways between the cortex and basal ganglia. The direct one goes from the striatum to the pallidum, and the indirect one loops within striatum-pallidum-subthalamic-pallidum. Both pathways reach the THA, but the damage to different pathways contributes to different outcomes. The impairment in direct pathway results in THA suppression, while damage in the indirect pathway leads to disinhibition and thalamic hyperactivity (Tekin and Cummings, 2002). Lu *et al*. found a significant GMV reduction and the shape deformities in left THA, which had connections with the frontal and temporal cortices and was found to be negatively correlated with depressive symptoms (Lu *et al*., 2016). Besides, the enhanced DTI properties of right anterior thalamic radiation tract was found in PPD, which connects the frontal lobe and dorsomedial THA in a direct pathway, recognized as a compensatory strategy to support negative emotions and challenge processing (Long *et al*., 2022). Thus, the over-suppression of THA_L in PPD versus MDD and HCs may contribute to emotional deregulation and could be a target for diagnostic assessments and therapies.

In the cortico-limbic circuit, ACC outputs messages to the ventral striatum and the substantia nigra, then projects to the medial THA. Finally, all the information is sent back to the ACC via the THA (Alexander *et al*., 1990). The sgACC is the core site of the ventral ACC, and is responsible for the negative emotional regulation through extensive connection with amygdala and orbital frontal cortex (OFC) (Etkin *et al*., 2011). For instance, the OFC/sgACC projects to the striatum, and then projects to the mediodorsal THA and ultimately back to the OFC. Dysfunction in this circuit is hypothesized to bias information processing in depression in such a way that depressed individuals selectively respond to and remember affectively negative information (Frodl *et al*., 2010). Notably, a previous PET study observed refractory depressive patients with hypermetabolism in the sgACC before treatment, and a reduced metabolism following the effective treatment (Mayberg *et al*., 2005). Besides, Shu *et al*. indicated the decreased FC between bilateral sgACC in young depressed patients with suicide attempts after treatment than pretreatment, indicating that the bilateral sgACC was relatively susceptible to treatment (Shu *et al*., 2022), which is also supported by the evidence from Yoshimura (Yoshimura *et al*., 2017). Taking all these findings together with our results, we speculate that the impaired function and reduced connections of the sgACC may be associated with a disregulate negative emotional expression and experience, and might be more sensitive in MDD than PDD.

Some limitations need to be noted in the current study. First, the sample size was relatively small, which may affect statistical power. Second, some potential confounding factors were not excluded in our study, such as fertility pattern (natural or assisted), natural or caesarean section, primipara or multipara, and cognitive function differences. A more delicately designed study with subgroup analysis is needed. Third, although resting-state fMRI is a widely used tool to examine brain functional alternations, further exploration of other measurements and other fMRI modalities (e.g. cortical thickness, task-based fMRI) are required in the subsequent studies to reveal more detailed information about the neuropathology of PPD and MDD in the future.

In conclusion, the present study revealed the common and disorder-specific neural mechanisms for PPD and MDD using voxel-wise analyses of low-frequency oscillation, local and global information integration for the first time. Both PPD and MDD patients presented hyperactivity left TP concerning emotion regulation in depression. PPD showed specific hypo-activities in the DMN (decreased fALFF in bilateral pMTG) and mentalizing network (decreased fALFF in left SMA), and hypo-connectivities within DMN (lower FC of pMTG with Pcu) and within the emotion regulation network (bilateral sgACC). MDD presented specifically enhanced global FCs in the right cerebellum. In addition, left sgACC and left THA showed significant differences in local FCs between any pair of groups of HCs, PPD, and MDD, proposing a different response to negative emotion stimulation. The specific neurofunctional markers for PPD and MDD found in this study may provide a better understanding of the underlying neural mechanism, and facilitate effective diagnosis and treatment strategies for PPD.
